# Characterization of early pathogenesis in the SOD1^G93A^ mouse model of ALS: part I, background and methods

**DOI:** 10.1002/brb3.143

**Published:** 2013-06-11

**Authors:** Sharon Vinsant, Carol Mansfield, Ramon Jimenez-Moreno, Victoria Del Gaizo Moore, Masaaki Yoshikawa, Thomas G Hampton, David Prevette, James Caress, Ronald W Oppenheim, Carol Milligan

**Affiliations:** 1 Department of Neurobiology and Anatomy, The Neuroscience Program and The ALS CenterWinston-Salem, North Carolina; 2 Department of Chemistry, Elon UniversityElon, North Carolina; 3 Mouse SpecificsBoston, Massachusetts; 4 Department of Neurology and the ALS Center, Wake Forest University School of MedicineWinston-Salem, North Carolina

**Keywords:** Axons, cytoplasmic vacuoles, glia, mega-mitochondria, mitochondria, motoneurons, motor function, NMJs

## Abstract

Charcot first described amyotrophic lateral sclerosis (ALS) in 1869; however, its causes remain largely unknown and effective, long-term treatment strategies are not available. The first mouse model of ALS was developed after the identification of mutations in the superoxide dismutase 1 (SOD1) gene in 1993, and accordingly most of our knowledge of the etiology and pathogenesis of the disease comes from studies carried out using this animal model. Although numerous preclinical trials have been conducted in the mutant SOD1 mouse models, the results have been disappointing because they did not positively translate to clinical trials. One explanation may be that current understanding of when and where pathogenesis begins is insufficient to accurately guide preclinical trials. Further characterization of these early events may provide insight into disease onset, help in the discovery of presymptomatic diagnostic disease markers, and identify novel therapeutic targets. Here, we describe the rationale, approach, and methods for our extensive analysis of early changes that included an ultrastructural examination of central and peripheral components of the neuromuscular system in the SOD1^G93A^ mouse and correlated these alterations with early muscle denervation, motor dysfunction, and motoneuron death. We also provide a discussion of published work to review what is known regarding early pathology in the SOD1 mouse model of ALS. The significance of this work is that we have examined early pathology simultaneously in both the spinal cord and peripheral neuromuscular system, and the results are presented in the companion paper (Part II, Results and Discussion). Our results provide evidence as to why a thorough characterization of animal models throughout the life span is critical for a strong foundation to design preclinical trials that may produce meaningful results.

## Introduction

Although the motor neuron disease, amyotrophic lateral sclerosis (ALS) was first described over 140 years ago in 1869 by the French neurologist Jean-Martin Charcot, its causes remain largely unknown and effective treatment strategies remain elusive (Bruijn and Cudkowicz 2006; Turner and Talbot 2008). The majority of cases are sporadic (sALS) while 10% are inherited in a dominant manner (fALS), but they are clinically indistinguishable. Pathological hallmarks include (a) spasticity and hyper-reflexia, reflecting dysfunction of “upper” motoneurons (MNs) in layer five of motor cortex, and (b) generalized weakness, muscle atrophy, fasciculations and paralysis, reflecting dysfunction and degeneration of MNs in the brainstem and spinal cord.

Several different chromosomal loci containing autosomal dominant mutations leading to adult fALS clinical onset had been identified (see Da Cruz and Cleveland 2011; Rademakers and van Blitterswijk 2013 for reviews). The first mutation identified associated with fALS was in the Cu/Zn superoxide dismutase (SOD1) gene that accounts for 20% of all forms of fALS (Boillée et al. 2006a). Mice and rats expressing mutant forms of human SOD1 develop progressive MN degeneration and clinical signs that closely mimic human ALS (Gurney et al. 1994) and accordingly most of our knowledge of the etiology and pathogenesis of the disease is from studies carried out over the past 15 years using these animal models (see Rothstein 2009 for review). Although numerous preclinical trials have been conducted in the mutant SOD1 mouse models, the results have been disappointing in that for various reasons (Scott et al. 2008), they did not positively translate to clinical trials (Benatar 2007).

In the SOD1^G93A^ mouse, symptom onset is reported to begin at approximately day 90, and one hallmark of this is that the animal fails to extend its legs when suspended by the tail. However, some studies report that symptom onset occurs closer to day 60 (Mancuso, Olivan et al. 2011; Mead et al. 2011; Gerber et al. 2012), and other studies (including the present study) suggest that more subtle behavior changes occur at even earlier time points (Amendola et al. 2004). Furthermore, pathological events such as fragmentation of the Golgi apparatus, vacuolization of mitochondria, deficits in axonal transport and endoplasmic reticulum (ER) stress are observed at early postnatal ages (Mourelatos et al. 1996; Stieber et al. 1998; Bendotti et al. 2001; Gonatas et al. 2006; Saxena et al. 2009). In many animal models of neuropathological conditions including ALS, there is increasing evidence that damage to axons and synapses often long precedes the activation of death pathways and the onset of clinical (i.e., functional) pathology (Coleman and Perry 2002; Raff et al. 2002; Medana and Esiri 2003; Palop et al. 2006; Gould and Oppenheim 2007; Saxena and Caroni 2007).

The research studies discussed above, together with many others in the literature have provided important insight into pathological events associated with disease in the mutant SOD1 mouse models for ALS. However, the question of when and where pathogenesis begins remains unanswered. Furthermore, the majority of studies focus on pathology in either the spinal cord or in the periphery in the axon or at the neuromuscular junction (NMJ). A systematic examination of pathological changes in both central and peripheral components of the neuromuscular system that occurs coincident with initial muscle denervation may provide insight into disease onset, help in the discovery of diagnostic disease markers, and identify novel therapeutic targets. Additionally, more detailed characterization as to when and where pathology begins has the potential to reevaluate previous preclinical trials as well as design more valid trials in the future. Here, we describe the approach for our study and review the literature regarding the early pathology in the SOD1 mouse model of ALS.

## Methods

### Animals

All animal experiments conformed to National Institutes of Health guidelines and were approved by the Wake Forest University Animal Care and Use Committee. Breeding pairs for *SOD1*^*G93A*^ [B6SJL-TgN (SOD1-G93A) 1Gur] mouse model were obtained from The Jackson Laboratory (Bar Harbor, ME). Nontransgenic wild-type (WT) females and *SOD1*^*G93A*^ males [B6SJL-TgN (SOD1-G93A) 1Gur] were bred to generate *SOD1*^*G93A*^ mice and nontransgenic WT littermates that were used in the experiments. Genotyping was performed with standard primers against mutant *SOD1* (Gurney et al. 1994; Truett et al. 2000). Experiments were blinded so that the individual performing the experiment did not know genotype until the analysis was complete.

### Statistical analysis

The specific statistical analysis used is noted below for each experimental approach. The Design Analysis Core at WFSM provided advice to the investigators, and the Design and Analysis Core performed select analyses. The number of animals used was based on experimental requirements for analysis and were chosen for a two-sided analysis of population means with an acceptable probability of a Type I error (*P*-value) of 0.05 or less and a probability of a Type II error of 0.05 or less. Statistical significance occurred when power was determined to be 80% or better and the *P*-value was equal to or less than 0.05.

### NMJ innervation

For counting innervated hind limb skeletal muscle NMJs, immunohistochemistry was performed on soleus and tibialis anterior (TA) muscles. Animals were transcardially perfused with 2% paraformaldehyde in 0.1 mol/L sodium phosphate buffer. The muscles were dissected, rinsed twice with phosphate buffered saline (PBS), and placed in 20% sucrose for at least 72 h at 4°C. The muscles were embedded in 20% sucrose:OCT (2:1) and cut at 30 μm on the cryostat. Antigen retrieval was achieved using a sodium dodecyl sulfate pretreatment (Brown et al. 1996) and the sections were stained for the vesicular acetylcholine transporter (VAChT; Santa Cruz Biotechnology, Santa Cruz, CA) and neurofilament light chain (NF-L; Millipore, Billerica, MA). Alexa-fluor 488 secondary antibodies were used for detection (green). Sections were also labeled with Alexa-fluor-alpha-bungarotoxin (α-BTX; Invitrogen, Eugene, OR; red) (Maeda et al. 2004). NMJs that exhibited an overlap of red and green were considered innervated, while those that exhibited only α-BTX expression were considered denervated. All NMJs in every 30 μm section were analyzed. The percentage of innervated NMJs was determined in each treatment group using previously established counting criteria (Gifondorwa et al. 2007). Statistical differences between WT and SOD1 groups were determined using unpaired *t*-test.

### MN and interneuron counts

Briefly, mice were transcardially perfused with PBS followed by Bouin's fixative. The lumbar region of the spinal cord was removed and embedded in paraffin. Twelve micrometer sections were cut and stained with a 5% thionin solution (Chu-Wang and Oppenheim 1978). Only healthy MNs were counted in every tenth section of the lumbar spinal cord using a well established reliable method that has been validated against an optical fractionator unbiased stereological counting method (Clarke and Oppenheim 1995). Healthy MNs are those that lie completely in the section with a nucleolus and normal MN morphology. Means and SEM were determined for each group. Statistical significance between the two groups was determined by a *t*-test with Bonferroni correction. Interneurons were counted in the same tissue using the same method employed for MN counts and statistical significance between the two groups was determined by a *t*-test with Bonferroni correction. (McKay and Oppenheim 1991; Clarke and Oppenheim 1995).

### Ventral root counts

The stereo dissector method was used to determine ventral root (VR) counts. Briefly, individual VRs were dissected and the tissue was processed for 1 μm plastic sections as described for electron microscopy (EM) processing. Images were acquired using 100×, oil immersion objective for each L3, L4, and L5 VR. A photomontage was created from the images and overlaid onto a grid template. VRs were counted in designated subdivisions (lower left in grid box). The area of the VRs was measured using Scion Image. An unpaired *t*-test was used to determine statistical differences between WT and SOD1 animals.

### Number of axons in intramuscular fasicles

TA muscles were obtained from mice that were perfused with 2% paraformaldehyde. After rinsing with PBS, muscles were placed in 20% sucrose overnight at 4°C and on the following day frozen in 20% sucrose:OCT (1:2). Muscles were sectioned at 70 mm and mounted on gelatin-coated glass slides. Intramuscular nerves and NMJs were analyzed using a silver-cholinesterase histochemistry (Pestronk and Drachman 1978) with a minor modification (10% silver nitrate solution). Quantitative data was obtained by counting the number of axons in intramuscular fascicles following the method described below in which large presumptive motor axons were included versus smaller sensory axons that were not included (Pun et al. 2006). Images were acquired using Scion Visicapture and the number of silver-esterase positive axons per intramuscular nerve branch were counted. A nerve branch was included when its individual axons could be followed to individual NMJs. For each muscle, 22–25 intramuscular nerve branches were counted. Statistical differences between WT and SOD1 groups were determined using unpaired *t*-test.

### Antibodies used

Well-characterized antibodies against cell- and synapse-specific markers were used for single- or double-labeling studies. A complete list of antibodies, immunogens, manufacturers, host species, dilutions used, and references are provided in Table 1. Immunostaining patterns in MNs, spinal cord, or muscle have been previously described for all antibodies and, in the current studies, all antibodies stained the appropriate cell types and showed the expected distribution.

**Table 1:** Antibodies used

Antigen	NIF ID	Antibody target	Source	Species	Concentration
Primary antibodies					
Calcitonin gene-related peptide (CGRP)	AB_2068655	Calca	Chemicon AB5920	Rabbit polyclonal	1:2000
Choline acetyltransferase (ChAT)	AB_2079751	ChAT	Chemicon AB144P	Goat polyclonal	1:100
Neurofilament light chain (NF-L)		Purified porcine NF-L	Chemicon/Millipore AB9568	Rabbit polyclonal	1:1000
Rab3A	AB_2177370		Synaptic Systems 107-102	Rabbit polyclonal	1:500
SV2 (pan)		Electric ray, synaptic vesicles	Dr. K Buckley, Harvard Medical School	Mouse monoclonal	1:500
Synaptophysin	AB_2198887	SYP	Synaptic Systems 101-002	Rabbit polyclonal	1:100
Vesicular acetyl choline transporter (VAChT)	AB_2301792	5/c 18a3	Santa Cruz Sc7717	Goat polyclonal	1:800

### Identification of MNs for ultrastructure analysis

**Figure 1:**
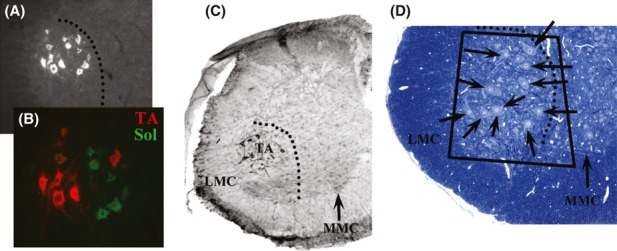
Illustration of approaches used to identify MNs. (A and B) The TA and soleus motor pools exhibit rostral caudal overlap when identified via retrograde labeling after muscle injections of fluorescently labeled Cholera toxin B subunit (CTB; A), but can be distinctly identified when soleus is injected with Alexa-Fluor 488 CTB (green in B) and TA is injected with Alexa-Fluor 555 CTB (red in B). (C) Immunohistochemistry was used to confirm CTB in MNs of mice where the TA was injected with CTB, confirming motor pool location. (D) Arrows in 1 mm toluidine blue stained sections (areas similar to those shown in A–C) from animals prepared for EM indicate αMNs typically used in the evaluation of synaptic type on MNs. Dotted line outlines the VH boundary, and the box indicates location of thin section. *X*-*Y* coordinates on the electron microscope were used to map the adjacent sections and located specific MNs. MNs, motoneurons; TA, tibialis anterior; VH, ventral horn.

To identify specific motor pools in the L3/L4 segments of the spinal cord for ultrastructural level, we initially used retrograde labeling with colloidal gold-labeled cholera toxin subunit B (CTB; List Biological Laboratories, Denver, CO); however, gold particles could not be definitely identified in MNs. Therefore, we identified the TA and soleus motor pools by retrograde labeling with alexa-fluor CTB (Invitrogen; Fig. 1A and B) and CTB-immunocytochemistry (Fig. 1C) at the light microscopic level. For immunohistochemical detection of CTB injected into the TA muscle to show the location of the TA motor pool along the rostral-caudal axis of the ventral horn (VH), free floating 150 μm vibratome sections were processed for immunohistochemistry to CTB (List Biological) using biotinylated secondary antibody and DAB to visualize reaction product (Vector Elite kit, Vector Laboratories, Burlingame, CA). From this initial experiment, the location of the two motor pools could be identified to target these MNs for examination of ultrastructure in separate material prepared for electron microscopy.

In separate animals prepared for electron microscopy, mice were deeply anesthetized with ketamine/xylazine and perfused intracardially with 100 mL of freshly made 2% glutaraldehyde, 2% paraformaldehyde in 0.13 mol/L sodium cacodylate buffer, pH 7.4, using a peristaltic pump at a flow rate of 10 mL/min. Perfused animals were kept at 4°C for 1–3 h, then spinal cords were carefully removed via dorsal laminectomy and place in fixative overnight at 4°C along with the various muscles. For VH, spinal cords were then embedded in 4% low temperature agarose cooled to 37°C, solidified on ice, cut on a vibratome at 250 μm, and collected serially. VH areas of interest were dissected using epi-illumination. The (L3–L4) area was determined by the position along the rostral-caudal length of the spinal cord and by motor pool appearance as determined in Figure 1. Specimens were then embedded in Araldite 502 using a Lynx processor. One micron sections and subsequent 700 Å thin sections were cut using an LKB ultramicrotome, then counterstained with either toluidine blue for 1 μm sections, or uranyl acetate in 100% methanol and subsequently lead citrate for thin sections, which were viewed and photographed digitally using a Zeiss EM 10 electron microscope (Carl Zeiss Microscopy, Hamburg, Germany). All analysis of ultrastructural features was performed on unaltered images that were collected directly from the electron microscope using the Orius EM high-resolution camera. Images shown in the figures were adjusted only for contrast.

Thin section maps were constructed using the X-Y stage controls of the electron microscope; subsequently the adjacent 1 μm section outline was superimposed onto this map using a camera lucida, and MNs marked (Fig. 1D). Those cells meeting α-MN criteria within the VH motor pool (>600 μm^2^, one or more C-terminals, nucleus with prominent nucleolus, abundant cytoplasm and organelles) were found on the electron microscope and photographed to create a montage of the cell surface at 16,000× magnification. MNs <600 μm^2^ were classified as γ-MNs (Friese et al. 2009; Shneider et al. 2009). Areas for all MNs examined were determined to confirm classification as α- or γ-MN.

For evaluation of ultrastructural changes in MNs in the L3–L4 segments of the spinal cord at day 30 of SOD1^G93A^ mice (*N* = 5, total of 38 MNs) were compared with findings from age-matched WT (*N* = 5, total of 48 MNs) animals. On the basis of the number of MNs labeled by retrograde transport and motor unit number estimation (MUNE) results in the literature, we estimate a total of 100 MNs that together compose the TA and soleus motor pools. We analyzed 5–10% of this population at the ultrastructural level. These same MNs were used to evaluate afferent synaptic input described below and mitochondria number and area. Mitochondria in the MNs identified in the high-resolution images were counted and areas measured using Image J software. Significant differences between WT and SOD1 groups was determined using unpaired *t*-tests.

Glial cells also identified based on their ultrastructural characteristics (Peters et al. 1991) in the same sections used for analysis of MNs were used for characterization of glial cells.

### Evaluation of afferent synapses on lumbar MNs

For examination of synapses on MN soma or distal dendrites, using the same segment levels as used for the MNs described above, 25–30 dendrites with synapses in the ventro-lateral white matter were identified and photographed at 16,000×. Only those synapses with a clear synaptic density and presynaptic vesicles were scored. Synapses were evaluated using classical criteria for identification of Type 1 or R (round), Type 2 or P (pleomorphic), or C-type synapses (Bodian 1964, 1966, 1970, 1972; Uchizono 1965, 1966; Hellström et al. 2003). Synapses were defined as having an identifiable synaptic density and/or fused vesicles at the presynaptic terminal (see Fig. 19 in accompanying paper, doi: 10.1002/brb3.142). Type 1 synapses (R) have ±50 nmol/L spherical vesicles, light ground substance, and were asymmetrical, with the postsynaptic density and synaptic cleft being larger than that of other synapse types (see Fig. 19 C and F in accompanying paper). Type 1 synapses are considered excitatory. Type 2 synapses (P) are considered inhibitory and have pleiomorphic or flattened vesicles, lighter symmetrical synaptic densities, and a thin synaptic cleft (see Fig. 19B, E, and F in accompanying paper). Type 3 synapses or C-terminals (C) are only found on αMNs and are cholinergic. These synapses overlie subsynaptic cisterns and organelles (rER), have a distinctive postsynaptic density that appears connected to ER, and have a high packing density of round or slightly flattened vesicles that were not as uniform as those found in Type 1 synapses (Fig. 19A, D, H, and G in accompanying paper). There are at least two other types of synapses (R bulbs or M terminals, and T terminals that exhibit electron dense bodies immediately adjacent to the postsynaptic density called taxi bodies); these synapse types are quite rare (<1%) and were not included in our analysis. Axodendritic synapses on the distal dendrites of MNs located in the white matter of the VH of these same animals were also assessed. Photographs were taken at 16,000 of the first 10–12 frames containing synapses found on dendrites within the white matter located ventro-laterally to the L3/L4 area used for MN synapse evaluation. These synapses were then blindly scored and the lengths of their dendrites measured using Image J. Statistical analysis was performed by the Design and Analysis Unit at WFSM. A mixed models approach was initially used to identify differences between WT and SOD1 while accounting for the repeated measures within each mouse and correlation between measures within each mouse. Statistical difference was determined using a least square means table.

To confirm changes in C-terminals, immunohistochemistry was performed. For the immunohistochemical quantification of C-terminals in the VH, cholinergic terminals surrounding MNs were identified using an antibody to vesicular acetylcholine transporter (VAChT; Table 1) and biotinylated secondary antibody and DAB to visualize reaction product (Vector Laboratories). The number of VAChT+ terminals surrounding at least 10 large α-MNs per animal, three animals per group, were then counted on an Olympus BX-50 microscope using 100× oil immersion and through focus. Statistical differences between WT and SOD1 groups were determined using unpaired *t*-tests.

### Characterization of VH white matter

For VH white matter measurements, axon number and size and glia number were determined in two 100× fields, one immediately ventral and one immediately lateral to the L3–L4 VH. Using Image J thresholding software under constant conditions, the number and mean size of axons was determined. In these same images the number of glia were also counted. For white matter width, five measures of width were made in a spoke-like fashion around the VH, then averaged and compared. Statistical differences between WT and SOD1 groups or between P14 and P30 were determined using unpaired *t*-tests.

### Identification and evaluation of ultrastructure of NMJs

Specific muscles were dissected from the mice used for ultrastructural analysis of the spinal cord described above and placed in fixative overnight at 4°C. Muscles were cut at 600 μm on a tissue chopper, then reacted for cholinesterase to reveal NJMs using Karnovsky's method with the following changes: sodium cacodylate buffer was used instead of PBS and the tissue was incubated for 1 h or until the endplates were visible under the dissecting microscope (Karnovsky, 1964). Specimens were then embedded in Araldite 502 using a Lynx processor. One micron sections and subsequent 700 Å thin sections were cut using an LKB ultramicrotome, then counterstained with either toluidine blue for 1 μm sections, or uranyl acetate in 100% methanol and subsequently lead citrate for thin sections, which were viewed with a Zeiss EM 10 electron microscope. Photographs were taken digitally using a Gatan ES 1000W or Orius camera. All analyses of ultrastructural features were performed on unaltered images that were collected directly from the electron microscope camera. Images shown in the figures were adjusted only for contrast to enhance visibility for the reader. Degenerative inclusions (vacuoles, tubulovesicles, autophagic vesicles, and dense core vesicles) have been reported to occur in aging or following ischemia in presynaptic NMJ terminals (Boaro et al. 1998; Tömböl et al. 2002). The criteria previously published in those papers were used when identifying these structures in our material.

Numerous measurements of structures of the NMJ including area of the axon terminal, junctional fold number, length and diameter, length of active zone, area and number of mitochondria were made using Image J software directly from the images acquired with the Orius EM high-resolution camera. The number of vesicles and of docked vesicles was also counted, and the number of vesicles/μm^2^ axon terminal and the number of docked vesicles/μm^2^ of active zone were derived. Presynaptic vesicles were characterized as “docked” vesicles if they were within one vesicle diameter (∼50 nm) of the presynaptic active zone (Schikorski and Stevens 1997, 2001; Tyler and Pozzo-Miller 2001; Rizzoli and Betz 2004). We defined active zone as the length of the presynaptic membrane that directly opposed the postsynaptic junctional folds. These criteria were applied for all material analyzed. A total of four WT and four SOD1 animals were analyzed with 5–10 NMJs evaluated for each muscle area (soleus, TA inside, and TA outside subcompartments) in each animal. Statistical analysis was performed by the Design Analysis Core at WFUSM using the restricted maximum likelihood, mixed effect (REML) model.

### Evaluation of spinal cord mitochondria function

Mitochondria were isolated from fresh livers and spinal cords of age-matched wild-type and *SOD1*^*G93A*^ mice by mechanical disruption followed by differential centrifugation as described previously (Del Gaizo et al. 2003; Pedrini et al. 2010). Mitochondria protein content was then determined by Lowry Assay (Biorad, Hercules, CA). For membrane potential assessment, mitochondria were diluted to 0.5 mg/mL in experimental buffer (125 mmol/L KCl, 5 mmol/L malate, 1 mmol/L potassium phosphate buffer, pH 7.4, 20 μmol/L EGTA/Tris, and 10 mmol/L Tris/MOPS, pH 7.4) and then incubated with 2 μmol/L tetramethylrhodamine, ethyl ester (Invitrogen) with and without 4 μmol/L CCCP (Sigma-Aldrich St. Louis, MO) and 0.2 μmol/L valinomycin (Sigma) for 10 min at room temperature. Each reaction was then spun at 10,000*g*, 4°C, for 5 min to pellet mitochondria and 50 μL of the supernatant added to separate wells of a 96 well plate (Corning) and fluorescence measured using excitation filter 485 nm (15 nm band pass) and emission filter of 590 nm (15 nm band pass). For ATP content and generation, the ATP Determination Kit (Invitrogen Molecular Probes, Eugene, OR) was used and modified according to Drew and Leeuwenburgh (2003).

### Axonal transport

Retrograde axonal transport was determined using previously published protocols (Ferri et al. 2003) with the exception that Alexa-Fluor-CTB was injected into the muscles rather than fluorogold. SOD1^G93A^ and WT mice were injected at P20 with Alexa-Fluor-CTB to label different MN pools. Because of the small size of muscles in preweaned mice P20 was the earliest time point when we were confident that injections were within individual muscles. Anesthesia induction using isofluorane took place using a vaporizer set at 5.0%, and mice were kept under anesthesia for the entire surgical procedure with the vaporizer between 1.5% and 2.0%. Once the TA and soleus muscle were exposed and identified, a volume of 4 μL Alexa-Fluor-555 CTB (5 μg/μL) was injected in the TA, and 2 μL Alexa-Fluor-488 CTB(5 μg/μL) was injected in the soleus muscle to identify both pools of fast-type and slow-type MNs, respectively. Muscle injection was carefully and slowly performed to avoid diffusion of the dye to adjacent muscles. A delay of several seconds was used prior to removing the needle from the muscle in order to minimize any dye reflux. Following the injection, surgical incisions were closed using a 4.0 silk-suture, and the mice were sacrificed between 0 and 96 h after injection.

Mice were perfused with 2% paraformaldehyde and the spinal cords removed. Spinal cords were cryoprotected in 20% sucrose, frozen in OCT medium, and sectioned at 20 μm using a cryostat (Leica Microsystems, Wetzlar, Germany). All spinal cord sections were collected, counterstained with DAPI, and MNs were examined under a regular epi-fluorescence microscope at 20×. The number of labeled MNs was determined. MNs were analyzed if they possessed a nucleus and exhibited Alexa-Fluor CTB label. MNs were not distinguished on the basis of intensity of CTB label. Statistical differences were determined using ANOVA followed by Tukey–Kramer post hoc test.

To determine MN size, P30 mice were injected as described above. Four days following injection, mice were perfused and tissue processed as described above. MNs were analyzed if they possessed a nucleus and exhibited Alexa-Fluor CTB label. MN area was determined using NIH Image J software. Statistical significance was determined using one-way ANOVA followed by Bonferrroni's multiple comparison test.

### Motor function

#### Gait dynamics

**Figure 2:**
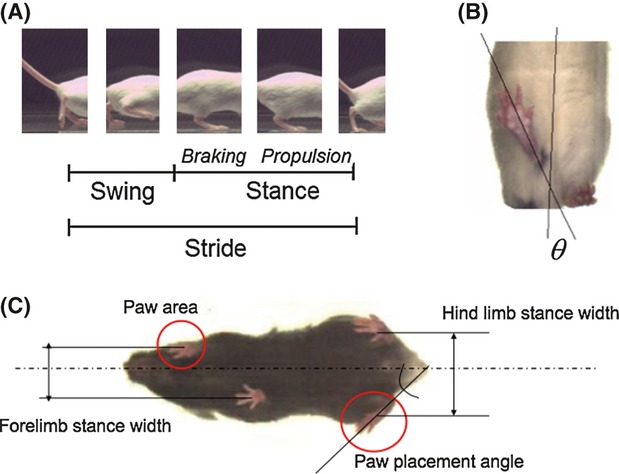
(A) Lateral view of a right hind paw during one *stride* depicting instances of time in *swing* and *stance*. Stance is comprised of *braking* and *propulsion*. (B) Paw Placement Angle is measured between the long axis through the hind paw and a line drawn through the center of the animal in its direction of motion. (C) Illustration of additional measurements for motor behavior. As described in the companion paper, the SOD1 mice exhibit a more closed hind paw placement angle, and less consistent step-to-step paw placement (increased paw placement angle variability) compared to WT mice. SOD1, superoxide dismutase; WT, wild type.

Gait dynamics were recorded using ventral plane videography, as previously described in detail (Hampton et al. 2004; Kale et al. 2004; Hampton and Amende 2010). Briefly, we used a motor-driven treadmill with a transparent treadmill belt (DigiGait Imaging System, Mouse Specifics, Inc., Quincy, MA). A high-speed digital video camera was positioned below the transparent belt to focus on the ventral view of subjects walking on the belt. An example of an SOD1 mouse being tested is shown (insert video). An acrylic compartment, *˜*7 cm wide by *˜*30 cm long and adjustable for length and width, was mounted on top of the treadmill to maintain the mice within the view of the camera. Digital video images of the underside of mice were recorded at *˜*150 frames per sec. Each image represents *˜*7 msec of locomotion; the paw area indicates the temporal placement of the paw relative to the treadmill belt. The color images were converted to their binary matrix equivalents and the areas of the approaching or retreating paws relative to the belt and camera were calculated throughout each stride. The plotted area of each digital paw print (paw contact area) imaged sequentially in time provides a dynamic gait signal, representing the temporal record of paw placement relative to the treadmill belt. Each gait signal for each limb comprises a stride duration that includes the stance duration when the paw of a limb is in contact with the walking surface, plus the swing duration when the paw of the same limb is not in contact with the walking surface. Stance duration was further subdivided into braking duration (defined as increasing paw contact area over time) and propulsion duration (defined as decreasing paw contact area over time). Stride frequency was calculated from the number of gait signals (see above) over time (Fig. 2a). Stride length was calculated from the following equation: speed = stride frequency *X* stride length. Stance widths and paw placement angles at full stance were obtained by fitting ellipses to the paws and determining the centers, vertices, and major axes of the ellipses. Forelimb and hind limb stance widths were calculated as the perpendicular distance between the major axis of the left and right fore paw images and between the major axis of the left and right hind paw images during peak stance. Paw placement angle was calculated as the angle that the long axis of a paw makes with the direction of motion of the animal during peak stance (Fig. 2b). Gait data were collected and pooled from both the left and right forelimbs, and the left and right hindlimbs.

Measures of stride-to-stride variability (gait variability) for stride length, stance width, and paw placement angle were determined as the standard deviation and the coefficient of variation (CV). The standard deviation reflects the dispersion about the average value for a parameter. CV, expressed as a%, was calculated from the following equation: 100× standard deviation/mean value. The maximal rates of change of area with respect to time, during the braking phase (+d*A*/d*t*_max_) and during the propulsion phase (−d*A*/d*t*_min_) of stance, were also determined.

Previous gait analysis of SOD1^G93A^ mice have indicated supranormal gait as compared with WT prior to neurodegeneration or the onset of gait disturbances at ∼13 weeks of age, when the animals walked horizontally at a walking speeds of 24 cm/sec and 36 cm/sec (Hampton and Amende 2010). In an effort to identify an earlier gait disturbance, we therefore challenged the animals with a more rigorous treadmill walking protocol in this study. The treadmill, walking compartment, and camera system were all pitched at an angle so that the animals walked up an incline of 15° (the angle of treadmill incline was based on published protocols [Brussee et al. 1997; Zhu et al. 2005; Whitehead et al. 2006]) and the walking speed was set to 40 cm/sec. Approximately 5 sec of video were collected for each walking subject to provide more than 20 sequential strides. Each subject was allowed to explore the treadmill compartment for ∼1 min with the motor speed set to 0, prior to treadmill videography with the animal walking uphill at 40 cm/sec. Only video segments in which the subjects walked with a regularity index of 100% were used for image analyses. The treadmill belt was cleaned between studies if necessary. Ventral view treadmill videography was performed weekly beginning when the mice were ∼30 days of age.

Data are presented as means *±* SE. ANOVA was used to test for statistical differences among WT and SOD1^G93A^ mice at each age. When the *F*-score exceeded *F* critical for *α* = 0.05, we used post hoc unpaired Student's two-tailed *t*-tests to compare group means. Gait indices between forelimbs and hindlimbs within groups were compared using paired Student's two-tailed *t*-tests. Differences were considered significant with *P* ≤ 0.05.

### Loaded grid test

**Figure 3:**
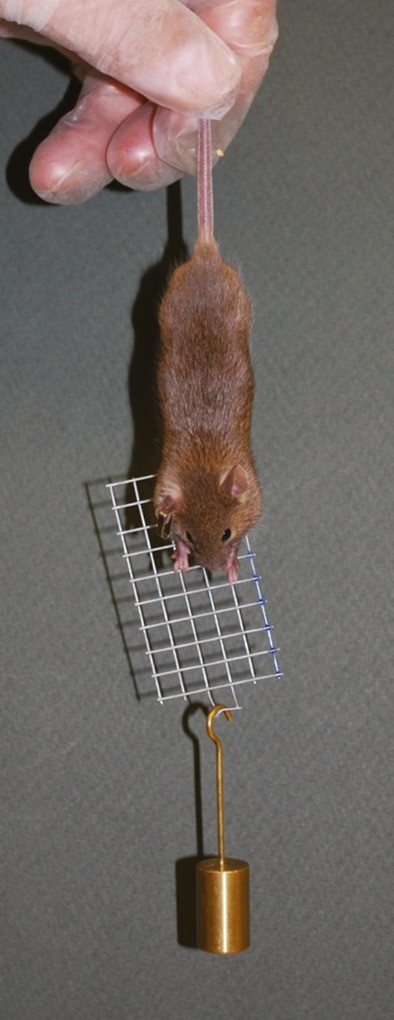
A wild-type (WT) mouse performing the loaded grid test is shown.

The loaded grid test described by Barnéoud et al. (1997) was used to assess forelimb muscle strength in mutant and WT mice (Fig. 3). Prior to the initiation of testing on P27 all mice received 2 days (P25 and P26) of handling (5 min/day) as well as pretraining with the unloaded grid. On P27, 28, and 29 each mouse was given two trials (separated by 10 min intertrial interval) with a 15 g weight and while suspended by the tail was allowed unlimited time to hold the loaded grid by the forelimbs before dropping it. On P30 each mouse was tested twice with each of four different weights (10, 20, 30 40 g in that order) and allowed only 30 sec per weight with a 15 sec interweight interval. There was a 10-min rest period between the two tests. Statistical differences between WT and SOD1 groups were determined using unpaired *t*-tests.

## Results

The results of the study are presented in the accompanying paper (doi: 10.1002/brb3.142).

## Literature Review

ALS is a debilitating neurodegenerative disease whose underlying causes and pathophysiology are not understood. As a result, there is no treatment that significantly ameliorates or delays the progression of the disease, and death resulting from respiratory failure occurs within 3–5 years from diagnosis. Many previous studies have focused on pathological events that occur coincident with or after symptom onset and MN degeneration. Identification and characterization of the earliest pathological changes in animal models can help distinguish initiating events from secondary events and provide insight into disease mechanisms resulting in MN dysfunction.

Research has centered on the MN cell body in the spinal cord as the key site of pathogenesis in ALS, but several studies have found that peripheral neuromuscular events may initiate the disease in terms of clinical symptoms, and supportive glial cells in the central nervous system (CNS) are also involved in disease pathology. Numerous ALS clinical trials have been unsuccessful, perhaps because the treatments are initiated too late in the course of the disease or because the targeted mechanism (e.g., cell bodies) are too far down the cascade of events that leads to motor neuron death. Therefore, it is critical to identify the site(s) in the nervous system where the first changes of ALS occur so that events earlier in the cascade can be targeted resulting in improved efficacy of treatment. Additionally, while muscle weakness, a prominent clinical symptom is thought to begin at the NMJ, pathology in both the peripheral and central nervous system may contribute to denervation and responses at both sites may prevent effective reinnervation and contribute to further MN dysfunction.

Several different chromosomal loci containing mutations leading to fALS have been identified with the second most common being mutations in the Cu/Zn SOD1 gene that account for 20% of all forms of fALS (Boillée et al. 2006a). Sporadic ALS and SOD1 mutant forms of fALS are clinically indistinguishable. Mice and rats expressing mutant forms of human SOD1 develop progressive MN degeneration and clinical signs that closely mimic human ALS (Gurney et al. 1994) and accordingly most of our knowledge of the etiology and pathogenesis of the disease is from studies carried out over the past 10 years using these animal models.

### Pathophysiology and histopathology of motor neuron disease in ALS mice

Motor neuron disease caused by mutant SOD1 in both humans and in animal models is due to toxicity of the mutant protein (gain-of-function), not to a loss-of-function of dismutase activity (Bruijn et al. 2004; Pasinelli and Brown 2006). Abnormal accumulation (aggregates/inclusions) of misfolded SOD1 in different cell types and cellular compartments is a likely mechanism for mutant SOD1 toxicity (Boillée et al. 2006a).

**Figure 4:**
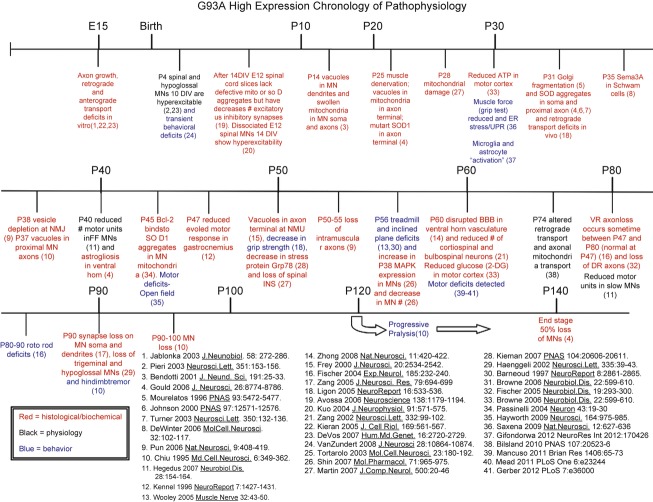
Chronology of pathophysiology in the SOD1^G93A^ mouse model of ALS. SOD1, superoxide dismutase; ALS, amyotrophic lateral sclerosis.

In mouse models of fALS and in histopathological studies of human autopsy material from both sporadic ALS and fALS cases, different cellular compartments of MNs appear to be primary or secondary sites of pathology. These include mitochondria, the Golgi apparatus, rough endoplasmic reticulum, neuromuscular synapses, MN axons. The neuron's response to oxidative stress, ER stress, or alterations in proteasome function may manifest in these pathological changes (Sánchez-Carbente et al. 2005; Fischer and Glass 2007; Boillée and Cleveland 2008; Cheroni et al. 2009). Physiological changes such as alterations in anterograde and retrograde axonal transport and hyperexcitotoxicity are also reported to occur. Both histological and physiological changes most likely lead to behavioral changes (see Fig. 4). Additionally the toxicity of mutant SOD1 involves other cell types besides MNs and therefore is at least partly noncell (MN) autonomous. For example, cell-specific deletion of mutant SOD1 in genetically altered mice has implicated microglia and astrocytes as contributors to the progression but not the onset of disease (Clement et al. 2003; Boillée et al. 2006b; Yamanaka et al. 2008a,b). In contrast, although selective mutant gene inactivation within MNs has shown that the timing (onset) of disease can be delayed (Yamanaka et al. 2008a), whether damage to cell types other than MNs can also affect disease onset is not clear. For example, alterations in astrocyte glutamate uptake have been hypothesized to contribute to disease pathology (see Vargas and Johnson 2010 for review), and microglial activation has been suggested to contribute to disease progression, but not onset (see Appel et al. 2011 for review). Interestingly, oligodendrocytes have recently been shown to be critical to MN survival via lactate transport, and their dysfunction may contribute to MN dysfunction and degeneration (Lee et al. 2012). Damage to other cell types including Schwann cells, interneurons, vasculature endothelial cells, and possibly muscle may also contribute to disease onset and progression in both familial and sporadic ALS patients and mouse models (Miller et al. 2006; Dobrowolny et al. 2008; Yamanaka et al. 2008b).

### Early events in the pathogenesis of motor neuron disease suggest synapse loss precedes MN degeneration

A cardinal feature of most developmental and adult onset neurodegenerative diseases, including motor neuron diseases such as ALS, is the death of specific population of neurons. Largely as a result of the progress made in elucidating the cellular and molecular mechanisms underlying neuronal death during development (Oppenheim et al. 2013), approaches aimed at ameliorating neurodegenerative disorders often focus on the manipulation of neuronal death pathways (Guégan and Przedborski 2003; Sathasivam and Shaw 2005). However, although neurodegenerative disorders involve the death of cell bodies as well as the loss of axons, dendrites and synapses, which of these occurs first and, more importantly, its relationship to disease onset (e.g., muscle weakness in ALS) are often not known. Additionally, because each of these neuronal compartments are interdependent, the first cellular compartment to be demonstrably affected may not be the site of the first molecular or biochemical events (Conforti et al. 2007).

Recent evidence derived from the study of animal models of various neuropathological conditions has revealed that damage to axons and synapses often long precedes the activation of death pathways and the onset of clinical (i.e., functional) pathology (Raff et al. 2002; Medana and Esiri 2003; Palop et al. 2006; Gould and Oppenheim 2007). In the case of mouse models of ALS, muscle denervation occurs months before MN death or disease onset (Frey et al. 2000; Fischer et al. 2004; Gould et al. 2006; Pun et al. 2006). Although there is a paucity of studies of this issue in humans, it appears that denervation may also precede disease onset in ALS patients (Tsujihata et al. 1984; Siklós et al. 1996; Aggarwal and Nicholson 2002; Fischer et al. 2004). In at least some neurodegenerative disease models with early onset of axon/synapse loss, including ALS, protecting cell bodies from death fails to alter disease progression or life span (Sagot et al. 1995; Kostic et al. 1997; Ferri et al. 2003; Chiesa et al. 2005; Libby et al. 2005; Gould et al. 2006; Suzuki et al. 2007). Clearly, these studies show that targeting the prevention of cell death per se is not likely to be the most effective therapy for treating these disorders. Rather, the loss of connectivity may be the most important contribution to the organism's disability and this aspect of neurodegenerative disease is a neglected potential therapeutic target. Indeed, the purpose of our study is to identify pathological changes that occur coincident or preceding NMJ denervation.

Denervation of NMJs by fast-fatigable (FF) MNs that innervate specific types of muscle fibers – myosin heavy chain (MyHC) Type IIB – in SOD1 fALS mice begins as early as P25 (Gould et al. 2006 (disease onset at P90-100), followed later by loss of NMJ innervation of Type IIa muscle fibers by fast-fatigue resistant (FR) MNs and lastly denervation by slow (S) MNs that innervate Type I/Ia muscle fibers (Pun et al. 2006). The early denervation of FF MNs is partially compensated for functionally by sprouting and reinnervation by FR and S MNs. However, eventually even these more resistant MN subtypes are unable to compensate at which point muscle weakness ensues (Hegedus et al. 2007), followed later by the loss (degeneration) of MN cell bodies.

### Age is a common feature of neurodegenerative diseases

While selected neuronal populations are affected in neurodegenerative diseases such as ALS, Alzheimer's and Parkinson's diseases, age is a common feature in all neurodegenerative diseases. Results from numerous studies suggest that there are common features across disease-specific populations including aggregation of misfolded proteins, altered proteasome activity and stress responses including ER stress, increased autophagy and mitochondrial changes noted above. Furthermore, patterns of resistance and susceptibility in NMJs in ALS mice are also observed in normally aging mouse muscles (Valdez et al. 2012). A recent review addressing the issue of selective neuronal vulnerability suggests that each neuronal population exhibits a stressor threshold, and proposes that each individual neuronal population is subjected to high levels of excitability resulting in increased levels of intracellular calcium (Saxena and Caroni 2011). For example, fast-fatigable muscle fibers, the first ones denervated in the SOD1 mouse model, are innervated by the most phasic subtype of MNs. Hyperexcitability and evidence of ER stress is detected specifically in MNs innervating fast-fatigable muscles prior to initial denervation further suggesting that these events serve as stressors that continue until the neuron reaches a threshold that jeopardizes its survival (Saxena and Caroni 2011). Nonetheless, many of the events associated with cellular stress often represent a protective response by the cell and therefore do not precipitate neuronal dysfunction, but rather prevent or delay the process (reviewed in Robinson et al. 2011; Gould and Milligan 2012).

### Axonal deficits in ALS

The events discussed above can lead to impairment of fast and slow axonal transport in vivo that has been well established in adult SOD1 mutant mice (Collard et al. 1995; Zhang et al. 1997; Warita et al. 1999; Williamson and Cleveland 1999; Sasaki et al. 2004; Ligon et al. 2005) and in human ALS patients (Bradley et al. 1983) deficits have even been reported in cultured embryonic MNs from SOD1 mice (Kieran et al. 2005; De Vos et al. 2007). Although the transport deficits in adult SOD1 mutant mice occur prior to disease onset, whether they occur before the onset of muscle denervation and thus represent a primary event or are secondary to axon/synapse loss or dysfunction is not known. The impairment of axonal transport in SOD1 mice has been attributed to appearance of neurofilament (NF) inclusions in mutant axons (Zhang et al. 1997), but mutations in transport proteins in the dynein/dynactin complex also occur (LaMonte et al. 2002; Hafezparast et al. 2003; Puls et al. 2003). The enrichment within neurons of mitochondria near sites of activity such as axons, dendrites, and presynaptic terminals indicates that mitochondrial localization may be the target of disease toxicity in fALS as well as in other neurodegenerative diseases involving distal-to-proximal axonal pathology (Gould and Oppenheim 2007). It is an attractive possibility that altered transport of either normal or defective mitochondria to and/or from MN presynaptic terminals contributes to neuromuscular denervation and MN disease (but see, Marinkovic et al. 2012).

### Mitochondrial pathology is prominent in ALS

Alterations in mitochondria morphology and function have been identified in both animal models and ALS patient material and have been proposed to contribute to disease pathology and progression (Schon and Przedborski 2011). Dysfunction in mitochondrial Ca^+2^ buffering, bioenergetics, fission, fusion, and transport occur in animal models of the disease (reviewed in Cozzolino et al. 2013). Defects in Ca^+2^ sequestration in mitochondria have also been identified presymptomatically in nerve terminals in SOD1^G93A^ and SOD1^G85R^ mice with increased mitochondria membrane potential following nerve stimulation that may contribute to dysregulation of transmitter release and eventual terminal degeneration. Increased Ca^+2^ in nerve terminals may activate calcium-dependent proteases such as calpains, that could preferentially affect MN terminals innervating fast-fatigable muscles (reviewed in Barrett et al. 2011). Although not unique to ALS, mitochondria dysfunction is thought to initiate or contribute to MN denervation and eventual degeneration. For example, olesoxime, a drug that targets mitochondrial pore opening, showed promise in preclinical studies, but unfortunately did not prolong patient survival (http://www.trophos.com), and dexpramipexole, a drug shown to improve mitochondria function, proved successful in preclinical trials and was well tolerated in ALS patients, but it did not exhibit efficacy in promoting function or survival in the Phase 3 clinical trial (http://www.biogenidec.com).

## Summary

**Figure 5:**
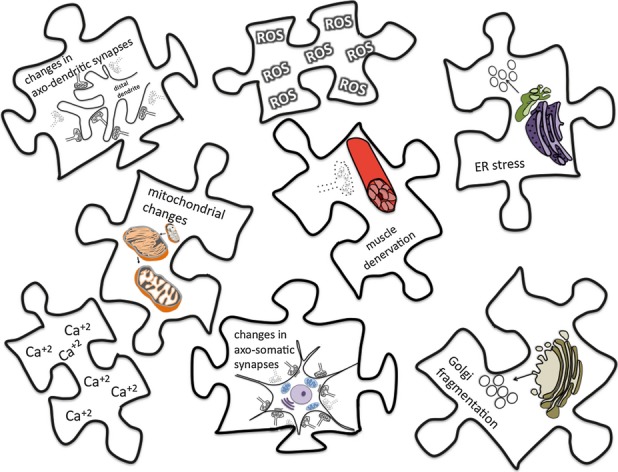
A summary diagram illustrating some of the pathological changes associated with mutant superoxide dismutase (SOD1) mouse models and putative patient disease progression. Research directed toward understanding how these events (puzzle pieces) are related and lead to clinical symptoms is critical to solving the puzzle for developing effective therapies.

Pathological events are well characterized in the ALS mouse models, but review of the literature fails to identify a specific initiating event that precipitates disease pathology. There is now a growing consensus in the field that the axon and synapses are the first cellular sites of degeneration, but there is still controversy over (1) whether axon and synapse loss is initiated autonomously at those sites or by pathology elsewhere (Bettini et al. 2007; Conforti et al. 2007; Gould and Oppenheim 2007) and (2) the specific molecular mechanisms mediating axon/synapse loss in ALS are largely unknown (Saxena and Caroni 2007). Mitochondrial morphological and functional changes are likely involved in disease pathology; however, alterations in synaptic input, axonal transport, ER stress, protein aggregates are also MN intracellular events that are associated with pathology. Extracellularly, reduced or altered vascular supply and glial activation may also contribute to disease pathology. Currently we have many pieces of a puzzle (Fig. 5), and understanding how they fit together to lead to muscle denervation, muscle weakness, and eventual loss of MNs, paralysis and death will provide targets for development of effective therapeutic strategies.

## Supporting Information

**Video S1.** Shown is an SOD1 mouse performing on the motor-driven, transpatent treadmill belt associated with the DigiGait Imaging System. Images of the movement patterns are collected with the camera mounted below the transparent treadmill and analyzed as described in the text and illustrated in Figure 2.
